# Pan-cancer profiling of FZD2 as a prognostic biomarker: integrative multi-omics analysis with experimental validation and functional characterization in gastric cancer

**DOI:** 10.3389/fphar.2025.1534974

**Published:** 2025-05-15

**Authors:** Sijiang Zhou, Da Li, Chao Quan, Zhu Yu, Yue Feng, Shengyu Wang, Yong Li, Tongtong Qi, Junqiang Chen

**Affiliations:** ^1^ Department of Gastrointestinal Surgery, The First Affiliated Hospital of Guangxi Medical University, Nanning, China; ^2^ Guangxi Key Laboratory of Enhanced Recovery after Surgery for Gastrointestinal Cancer, Nanning, China; ^3^ Guangxi Clinical Research Center for Enhanced Recovery after Surgery, Nanning, China; ^4^ Guangxi Zhuang Autonomous Region Engineering Research Center for Artificial Intelligence Analysis of Multimodal Tumor Images, Nanning, China; ^5^ Department of Surgery, University of Michigan Medical School, Ann Arbor, MI, United States; ^6^ Department of Urology, Xiangya Hospital, Central South University, Changsha, Hunan, China; ^7^ Pediatric Surgery, The First Affiliated Hospital of Guangxi Medical University, Nanning, China

**Keywords:** Fzd2, STAD, pan-cancer, drug sensitivity, immune infiltration

## Abstract

**Background:**

Frizzled class receptor 2 (FZD2), is a critical protein in the Wnt signaling pathway, which plays significant roles in various cancers. However, its role in cancer progression, prognosis, and diagnosis remains largely unexplored. This study investigates the correlation between FZD2 expression and clinical outcomes, as well as its underlying molecular mechanisms in pan-cancer.

**Methods:**

A comprehensive bioinformatic analysis was performed using pan-cancer data from The Cancer Genome Atlas (TCGA), which included 33 cancer types. Gene set enrichment analysis (GSEA) was conducted to explore functional pathways, while a protein-protein interaction (PPI) network was constructed to further elucidate the role of FZD2 in tumor biology. The relationship between FZD2 expression and immune cell infiltration across 22 categories was assessed using CIBERSORT. Additionally, single-cell analysis was employed to examine FZD2 expression levels across different cell types. To investigate the functional impact of FZD2, loss-of-function experiments were carried out in gastric cancer cell lines using siRNA-mediated knockdown. Subsequent assays, including Polymerase Chain Reaction (PCR), Western blotting (WB), Cell Counting Kit-8 (CCK8), Flow Cytometry, wound healing, and transwell migration and invasion assays, were performed to assess cellular responses. A subcutaneous gastric cancer xenograft model was established in nude mice to investigate the effect of FZD2 knockdown on tumor growth *in vivo*.

**Results:**

Our analysis revealed significant upregulation of FZD2 in multiple malignancies, including stomach adenocarcinoma (STAD), bladder cancer (BLCA), and cholangiocarcinoma (CHOL). FZD2 expression was correlated with various cancer characteristics, including stemness score, matrix score, immune score, tumor mutational burden (TMB), microsatellite instability (MSI), RNA modification genes, and drug sensitivity. Notably, FZD2 was associated with altered sensitivity to several anticancer agents, suggesting its role in modulating treatment responses. FZD2 knockdown was demonstrated by both *in vitro* and *in vivo* experiments to suppress tumor cell proliferation, migration, and invasion in gastric cancer cell lines, indicating its critical role in tumor progression. Furthermore, FZD2 exhibited significant correlations with other Wnt pathway genes (e.g., Wnt2, Wnt4, Wnt5B), indicating a complex interaction network contributing to tumorigenesis.

**Conclusion:**

FZD2 is widely upregulated in various tumor types, with its expression closely associated with key clinical outcomes, including overall survival, disease-specific survival, disease-free interval, as well as tumor mutations, drug sensitivity, immune cell infiltration, and immunotherapy-related biomarkers such as TMB and MSI. These findings highlight the pivotal role of FZD2 in cancer prognosis and treatment, offering potential for novel therapeutic approaches and the development of personalized medicine strategies in oncology.

## 1 Introduction

Cancer remains one of the most pressing global health challenges, characterized by heterogeneous pathogenesis, treatment resistance, and dismal prognosis across diverse malignancies. The development of pan-cancer biomarkers capable of predicting cancer progression, guiding precision treatment, and stratifying patients remains a critical unmet need.

Among the molecular regulators with pan-cancer relevance, the Frizzled (FZD) family of G-protein coupled receptors (GPCRs) has emerged as a pivotal node in oncogenic signaling. Comprising ten members (FZD1-FZD10), these receptors mediate Wnt ligand binding and pathway activation, which play fundamental roles in cell proliferation, differentiation, and epithelial-mesenchymal transition (EMT) (Zhang et al., 2018; Zhang and Wang, 2020). Dysregulation of FZD expression, particularly its overactivation in various cancers, has been linked to Wnt/β-catenin pathway hyperactivation, driving tumorigenesis, metastasis, and drug resistance (Dihlmann and von Knebel Doeberitz, 2005; MacDonald and He, 2012).

FZD2, a critical member of the Frizzled family, functions as a novel tumor marker and a pivotal receptor in Wnt signaling pathways, with specialized roles in non-canonical Wnt signal transduction. Its high expression in glioma and gastric cancer correlates with tumor growth, metastasis, and therapy resistance (Qi et al., 2017; Huang et al., 2019). Extensive studies further demonstrate that FZD2 overexpression induces oncogenic phenotypes across multiple cancers, including glioma and gastric cancer (Tomizawa et al., 2015; Bian et al., 2016; Tomizawa et al., 2016; Fu et al., 2020). Despite these associations, systematic characterization of FZD2 through multi-omics approaches remains scarce, particularly regarding its interactions with the tumor microenvironment (TME), genomic instability, and drug response landscapes.

**FIGURE 1:**
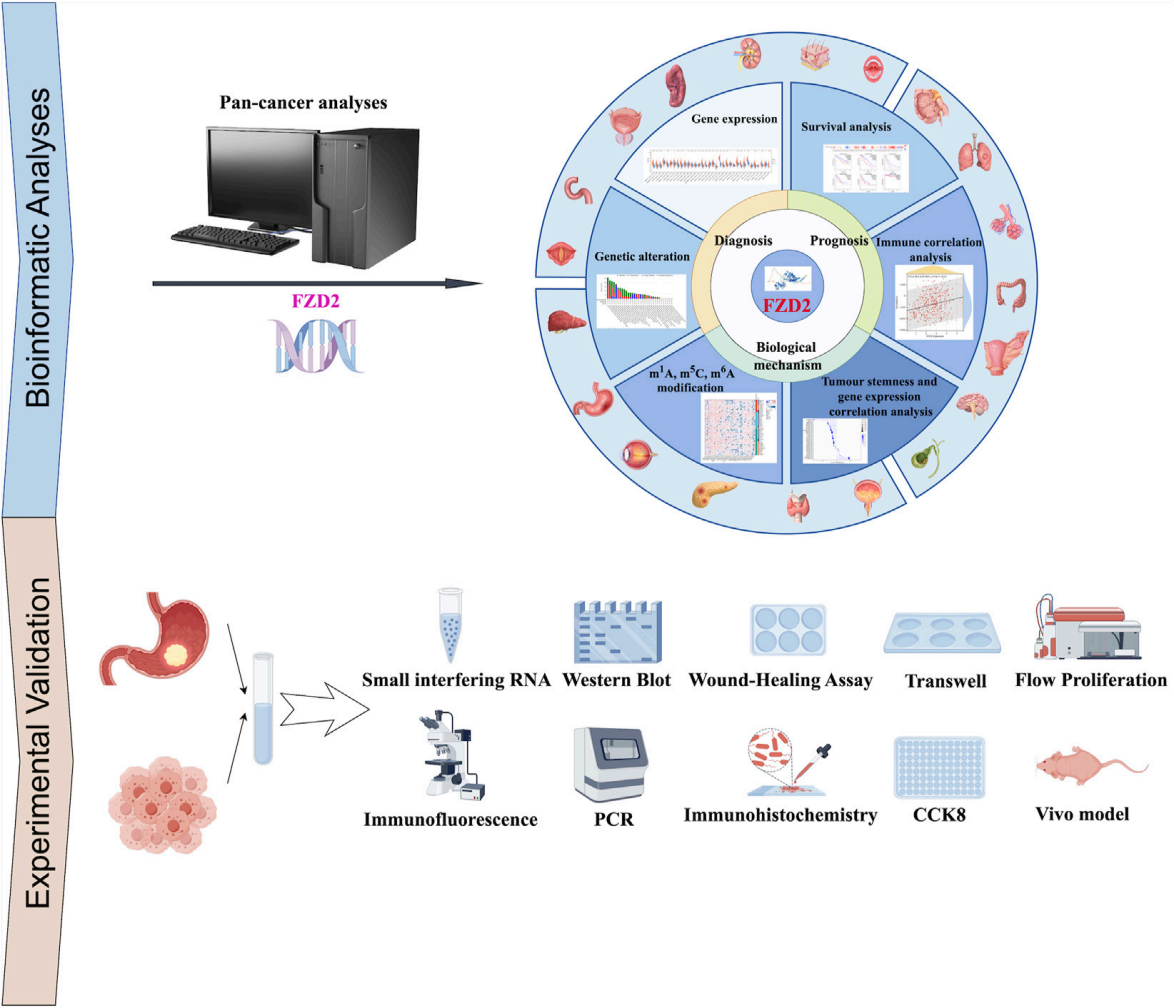
Workflow of the study. The study workflow outlines the retrieval of batch-corrected and normalized expression data, along with clinical information from The Cancer Genome Atlas (TCGA) and targeted datasets. A comprehensive pan-cancer analysis of FZD2 was performed, encompassing gene expression, protein expression, survival status, mutation analysis, DNA methylation, N6-methyladenosine (m6A) modification, gene set enrichment analysis (GSEA), receiver operating characteristic (ROC) analysis, and immuno-correlation analysis. To experimentally validate FZD2 expression, tissue microarrays from gastric cancer patients were utilized. Functional investigations of FZD2 in gastric cancer cell lines included PCR, cell proliferation assays, flow cytometry, immunofluorescence, colony formation, co-immunoprecipitation, and Western blotting.

To address this critical knowledge gap, this study employs a rigorous multi-omics data analysis framework using The Cancer Genome Atlas (TCGA) and Gene Expression Omnibus (GEO) cohorts to investigate FZD2’s expression patterns, prognostic value, immune microenvironment correlations, and drug sensitivity across pan-cancers. Our analysis identifies FZD2 as a prognostic biomarker associated with genomic instability (e.g., copy number variations in Wnt pathway genes), immune microenvironment remodeling (e.g., T cell exhaustion and M2 macrophage polarization), and unfavorable clinical outcomes. A particular focus on gastric adenocarcinoma – a major cause of global morbidity and mortality – reveals that FZD2 overexpression activates oncogenic pathways (Wnt/β-catenin and Notch) and induces EMT, thereby promoting metastasis and chemoresistance. This mechanism aligns with cross-talk between FZD2 and Notch signaling observed in breast cancer (Yin et al., 2020; Tuluhong et al., 2021), underscoring its potential role in gastric cancer progression. Building on these findings, we integrate *in vitro* and *in vivo* experimental models with bioinformatics tools to explore FZD2’s regulatory networks. The workflow of this study was shown in Figure 1. Our transdisciplinary approach not only establishes FZD2 as a tissue-specific pan-cancer biomarker with dual utilities (prognostic and therapeutic) but also demonstrates how integrated multi-omics strategies can uncover actionable targets for precision oncology.

## 2 Materials and methods

### 2.1 Data collection and pre-processing

RNA-seq data (raw counts, TPM), somatic mutations, survival records, and clinical annotations were acquired from the UCSC Xena Pan-Cancer Atlas (https://xena.ucsc.edu/) (Goldman et al., 2020). Initial data were processed by converting read counts to transcripts per million (TPM) and then normalised to log2 (TPM+1). Only samples with relevant clinical data were included to form the final dataset for further analysis. Normal tissue expression data were sourced from GTEx (https://www.gtexportal.org/home/), and FZD2 expression across immune cell types was examined using TISCH2 (http://tisch.comp-genomics.org/home/) (Sun et al., 2021). Single-cell datasets focusing on stomach adenocarcinoma (STAD) were obtained from the Gene Expression Omnibus (GEO) (https://www.ncbi.nlm.nih.gov/geo/), specifically from datasets GSE134520 and GSE167297 (Zhang et al., 2019; Jeong et al., 2021).

### 2.2 Differential expression analysis

Transcriptomic disparities between malignant and normal tissues were systematically evaluated across 33 cancer types using TIMER2.0 (http://timer.cistrome.org/) (Li et al., 2020). For FZD2-specific analysis, we integrated GTEx and TCGA datasets through SangerBox platform (http://vip.sangerbox.com), applying quantile normalization with variance stabilizing transformation (Chen et al., 2024). In a pan-cancer analysis, FZD2 expression was assessed in tumor samples versus their corresponding adjacent normal tissues. Additionally, immunohistochemistry data for FZD2 in gastric cancer and normal stomach tissues were obtained from the Human Protein Atlas (HPA) (https://www.proteinatlas.org/).

### 2.3 Analysis of FZD2 diagnostic value and expression by clinical stage

The receiver operating characteristic (ROC) curves were constructed using the pROC package (v1.18.0) in R to evaluate FZD2’s diagnostic potential. Bootstrap resampling (1,000 iterations) was applied to calculate the AUC with 95% confidence intervals (CI), while optimal cutoff thresholds were determined *via* Youden index (sensitivity/specificity reported). For clinical staging analysis, FZD2 expression across TNM stages (AJCC 8th edition) was compared using Kruskal-Wallis tests with Dunn’s *post hoc* correction, implemented through SangerBox (v3.8, http://vip.sangerbox.com).

### 2.4 Survival prognosis analysis

Survival analyses were conducted using GEPIA2 (http://gepia2.cancer-pku.cn/#index) with TCGA pan-cancer data. Patients were stratified by median FZD2 expression. Log-rank tests with Benjamini-Hochberg correction (FDR<0.1) assessed overall survival (OS) and disease-free survival (DFS) differences. In gastric cancer patients, associations between FZD2 mRNA expression and prognostic metrics—including first progression (FP), OS, and post-progression survival (PPS)—were assessed using Kaplan-Meier (https://kmplot.com/analysis/) analysis through the K-M plotter tool. Hazard ratios (HR) and 95% confidence intervals (CI) were calculated to quantify risk.

### 2.5 Genetic mutation and RNA modification analysis

Genomic alterations and RNA modifications of FZD2 across various cancers were analyzed using TCGA data in cBioPortal (https://www.cbioportal.org/). The analysis of FZD2 alterations included copy number amplifications, mRNA upregulation, missense mutations of uncertain significance, and deep deletions. Associations between FZD2 and RNA modifications (m^6^A, m^5^C, m^1^A) were evaluated using Pearson’s correlation in SangerBox (Wang et al., 2024).

### 2.6 Protein-protein interaction (PPI) network analysis

A protein-protein interaction (PPI) network for FZD2 was built in STRING (https://cn.string-db.org/), applying a minimum interaction score threshold of 0.4. Active interaction sources included text mining, databases, co-occurrence, neighborhood, experimental data, co-expression, and gene fusion (Szklarczyk et al., 2023).

### 2.7 Analysis of FZD2 expression in tumor mutational burden (TMB) and microsatellite instability (MSI)

Mutation data were sourced from UCSC Xena (https://xena.ucsc.edu/) and processed through VarScan2 for variant aggregation and masking. The correlation between FZD2 expression levels and both microsatellite instability (MSI) and tumor mutational burden (TMB) was evaluated using Spearman’s rank correlation.

### 2.8 Correlation analysis between immune cell infiltration and FZD2 expression

To investigate the role of the tumor microenvironment (TME) in cancer treatment, we analyzed the correlation between FZD2 expression and immune scores using the ESTIMATE algorithm. Additionally, infiltration levels of 22 immune cell types were quantified through the CIBERSORT algorithm (Yoshihara et al., 2013).

### 2.9 Drug sensitivity analysis

Drug sensitivity was evaluated using the R package pRRophetic (version 1.8.0), which analyzed FZD2 mRNA expression alongside drug response data. Based on the median expression cutoff, lesions were classified into high- and low-FZD2 expression groups, and differences in drug sensitivity between these groups were assessed using the Wilcoxon test, with results visualized in violin plots.

### 2.10 Sample collection

Histopathological analysis confirmed STAD diagnosis, after which tissue samples were collected from the First Affiliated Hospital of Guangxi Medical University. Samples were stored at −80°C immediately following surgery, with ethics approval granted by the hospital’s ethics committee.

### 2.11 Cell culture

Gastric cancer cell lines (AGS, HGC-27, MKN-28, and SNU-216) and normal gastric cells (GES-1) were obtained from Prosperity Life Sciences Ltd. (Wuhan, China). The cells were cultured in either Ham’s F12 medium (Gibco, China) or RPMI 1640 medium (Gibco, China), each medium supplemented with 10% fetal bovine serum (FBS, BI, Israel) and 1% penicillin-streptomycin solution (Wisent, Canada). Cells were maintained at 37°C in a humidified incubator with 5% CO_2_.

### 2.12 *In Vivo* tumor xgnograft model

FZD2-knockout (KO) AGS and HGC-27 gastric cancer cells (5 × 10^6^ cells/mouse) were resuspended in 100 μL sterile PBS and mixed at 1:1 (v/v) with Ceturegel^®^ matrix gel (Yeason, China). The mixture was injected subcutaneously into the flank area of 6–7 weeks old male BALB/c-nude mice (n = 3 per group). The mice were housed under specific pathogen-free (SPF) conditions in a temperature-controlled environment with a 12-h light/dark cycle. Tumor growth was monitored, and when the tumor volume reached or exceeded 1 × 10^3^ mm^3^, the mice were euthanized to conclude the experiment. The subcutaneous tumors were subsequently excised, weighed, and photographed. All animal experiments were conducted with ethical approval from the Ethics Committee of the First Affiliated Hospital of Guangxi Medical University.

### 2.13 Reagents and cell transfection

Small interfering RNAs (siRNAs) targeting FZD2 and control siRNAs were purchased from Hanbio (China). Transient transfection of siRNAs was carried out using Lipo3000 (Invitrogen, United States). Protein and RNA were harvested from the cells 48 h after transfection. The sequences of the siRNAs used were: si-NC forward, UUC​UCC​GAA​CGU​GUC​ACG​UTT, si-NC reverse, ACG​UGA​CAC​GUU​CGG​AGA​ATT, FZD2-si1 forward, CCU​AUC​UCA​GCU​ACA​AGU​UUC, FZD2-si1 reverse, AAC​UUG​UAG​CUG​AGA​UAG​GAU, FZD2-si2 forward, CCA​CGU​ACU​UGG​UAG​ACA​UGC, FZD2-si2 reverse, AUG​UCU​ACC​AAG​UAC​GUG​GUG, FZD2-si3 forward, CGG​UCU​ACA​UGA​UCA​AAU​ATT FZD2-si3 reverse, UAU​UUG​AUC​AUG​UAG​ACC​GTT.

### 2.14 RNA extraction and quantitative PCR (qPCR)

Total RNA was extracted from cells and tissues using NucleoZOL^®^ (Cat. No. 740404.200, MNG, Germany) following the manufacturer’s instructions. Q-PCR was conducted in triplicate on the Bio-Rad iCyCler real-time PCR7500 system with SYBR Green. Relative FZD2 levels were normalized to *ACTB* expression and calculated using the 2^−ΔΔCt^ method. The primer sequences were: ACTB forward, 5ʹ-TGG​CAC​CCA​GCA​CAA​TGA​A-3ʹ; ACTB reverse, 5ʹ-CTA​AGT​CAT​AGT​CCC​CTA​GAA​GCA-3ʹ; FZD2 forward, 5ʹ-GTG​CCA​TCC​TAT​CTC​AGC​TAC​A-3ʹ; FZD2 reverse, 5ʹ-CTG​CAT​GTC​TAC​CAA​GTA​CGT​G-3ʹ (Bong et al., 2024).

### 2.15 Western blotting

Cells and tissues were lysed using RIPA buffer (R0010, Solarbio, China) supplemented with PMSF (P0100, Solarbio, China). Lysates were separated by 10% SDS-PAGE and transferred to PVDF membranes (IPVH00010, Millipore, United States). After blocking and washing, the membranes were incubated with HRP-conjugated goat anti-rabbit IgG (SA00001-2, Proteintech) or HRP-conjugated goat anti-mouse IgG (SA00001-1, Proteintech). Anti-FZD2 antibody (TP72253) was purchased from Abmart and anti-α-tubulin antibody (66031-1-Ig) was purchased from Proteintech (Begum et al., 2022).

### 2.16 Wound healing assay

After 48 h of transfection, AGS and HGC-27 cells were plated in 6-well plates and incubated for 24 h, a scratch was made using a 200 μL pipette tip. After rinsing with phosphate-buffered saline, and the basal medium was added, images were taken at 0 and 48 h. The wound area was quantified using ImageJ software version 1.52 (NIH, United States) (Martinotti and Ranzato, 2020).

### 2.17 Cell proliferation, invasion, and migration assays

After 48 h of transfection, the cells were seeded into 96-well plates, and cell proliferation was evaluated using the Cell Counting Kit-8 (CCK-8) assay (A311-01, Vazyme, China). Optical density at 450 nm (OD450) was measured at 24, 48, and 72 h to assess cell viability. The Matrigel (Yeason, China) was removed from −20°C storage and thawed overnight at 4°C. It was then diluted in medium at a 1:9 ratio. For the invasion assay, 100 μL of the diluted Matrigel was added to the upper chamber of the Nunc™ polycarbonate cell culture inserts and incubated at 37°C to allow the formation of a matrix membrane. Following incubation, the inserts were hydrated with medium for 1 h before cell seeding. This step was omitted for the migration assay. Cell migration and invasion were evaluated using Transwell chambers with 8-μm pores (Corning, United States). Cells were seeded in serum-free medium in the upper chamber and cultured with 10% serum medium in the lower chamber. After fixation in 4% paraformaldehyde, the cells were stained with 0.1% crystal violet (Adan et al., 2016).

### 2.18 Apoptosis assay

Cells were digested with Accutase, collected, and centrifuged for 5 min. The supernatant was discarded, and the cells were washed once with PBS, centrifuged again for 5 min, and the supernatant was removed. Following an additional PBS wash and centrifugation, the supernatant was discarded once more. The cells were then resuspended in 100 μL of diluted 1× Annexin V Binding Buffer, followed by the addition of 2.5 μL of PI reagent (1 μg/mL) and 2.5 μL of Annexin V-FITC reagent. The mixture was gently vortexed to ensure thorough mixing and incubated at room temperature in the dark for 20 min. After incubation, 500 μL of PBS was added for final suspension before flow cytometric analysis using a BD FACSVerse flow cytometer (BD Biosciences, Canada) (Telford, 2018).

### 2.19 Statistical analysis

GTEx and TCGA data were analyzed using the Student’s t-test. The relationship between FZD2 expression and immune cell abundance was evaluated with Spearman’s correlation in TIMER2.0. All analyses were performed in R (version 4.2.1), with statistical significance defined as *p* < 0.05.

## 3 Results

### 3.1 mRNA expression of FZD2 in different types of human cancers

**FIGURE 2:**
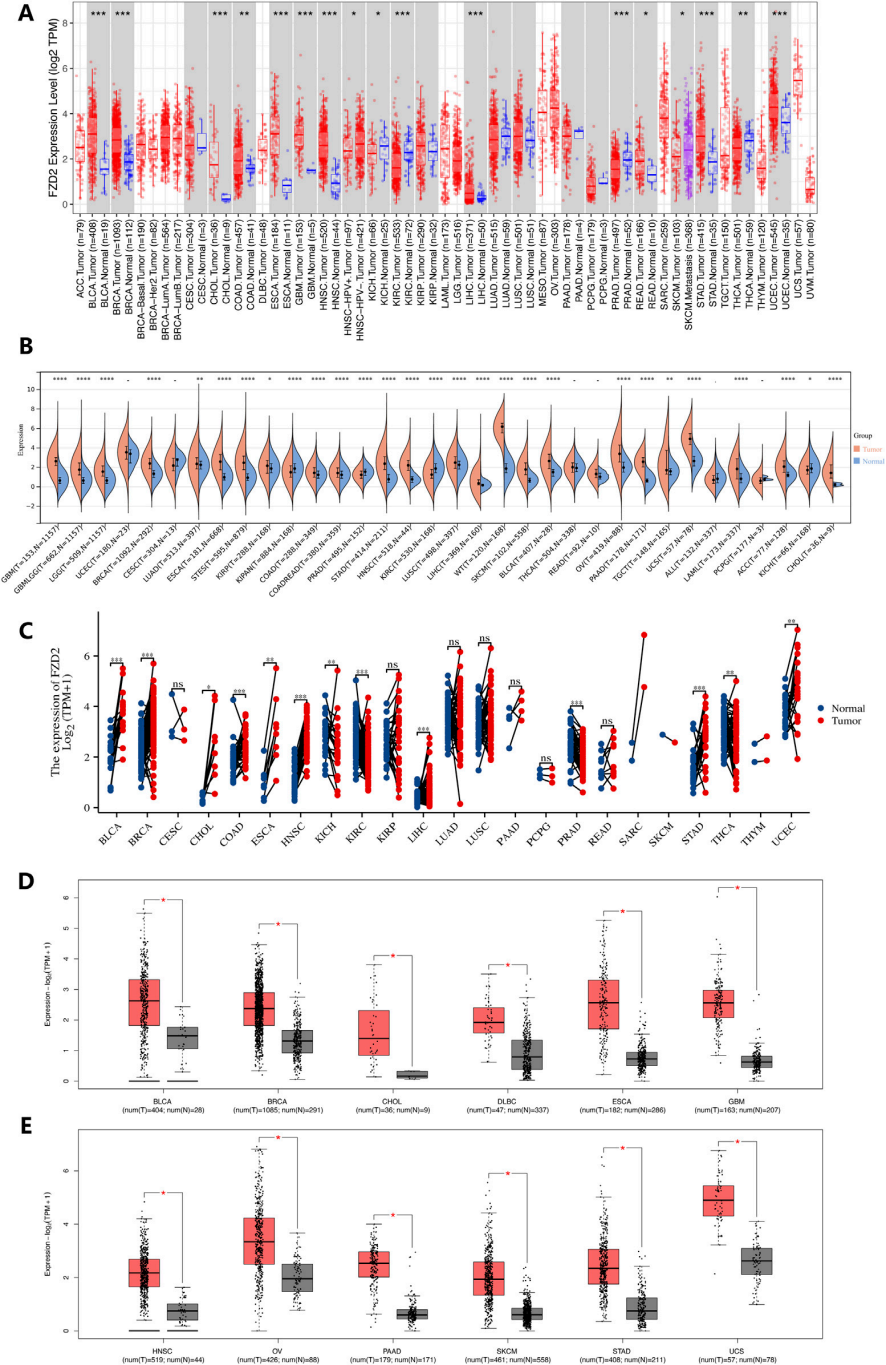
Gene expression analysis. **(A)** FZD2 expression across various cancer types and specific cancer subtypes analyzed using TIMER2. **(B)** Differential FZD2 mRNA levels in different tumors and normal tissues, based on TCGA and GTEx data. **(C)** Comparison of FZD2 expression in tumor samples and matched normal tissues from multiple cancer types, as obtained from TCGA. **(D,E)** FZD2 expression in BLCA, BRCA, CHOL, DLBC, ESCA, GBM, HNSC, OV, PAAD, SKCM, STAD, and UCS tumors from TCGA, with normal tissue data from the GTEx database used for comparison. *P < 0.05, **P < 0.01, ***P < 0.001, ****P < 0.0001.

As illustrated in Figure 2A, differences in FZD2 mRNA expression between tumor and normal tissues were analyzed using TIMER (TCGA RNA-seq data), revealing FZD2 overexpression in several cancer types. Specifically, FZD2 expression was significantly elevated in bladder cancer (BLCA), breast cancer (BRCA), cholangiocarcinoma (CHOL), colon adenocarcinoma (COAD), esophageal carcinoma (ESCA), glioblastoma (GBM), head and neck squamous cell carcinoma (HNSC), liver hepatocellular carcinoma (LIHC), rectal adenocarcinoma (READ), stomach adenocarcinoma (STAD), and uterine corpus endometrial carcinoma (UCEC) relative to normal tissues. In contrast, FZD2 expression was notably reduced in kidney chromophobe (KICH), kidney renal clear cell carcinoma (KIRC), prostate adenocarcinoma (PRAD), and thyroid carcinoma (THCA). Further analysis of cancer and adjacent normal tissues using the GTEx database revealed significant differences in FZD2 expression in GBM, lower-grade glioma (LGG), lung adenocarcinoma (LUAD), testicular germ cell tumors (TGCT), skin cutaneous melanoma (SKCM), ovarian serous cystadenocarcinoma (OV), pancreatic adenocarcinoma (PAAD), and other cancers. No significant differences in FZD2 expression were observed in UCEC, cervical squamous cell carcinoma (CESC), THCA, READ, acute lymphoblastic leukemia (ALL), or pheochromocytoma and paraganglioma (PCPG) (Figure 2B). Moreover, Figure 2C further demonstrates significant upregulation of FZD2 in tumor tissues across a range of cancers, including BLCA, BRCA, CHOL, COAD, ESCA, HNSC, LIHC, STAD, and UCEC, with lower expression observed in KICH, KIRC, PRAD, and THCA. GEPIA analysis corroborated these findings, showing significant elevation of FZD2 in tumor tissues compared to normal tissues in multiple cancer types, including BLCA, BRCA, CHOL, DLBC, ESCA, GBM, HNSC, OV, PAAD, SKCM, STAD, and UCS (*P* < 0.05) (Figures 2D,E).

### 3.2 Correlation between FZD2 expression and clinical staging in pan-cancer

**FIGURE 3:**
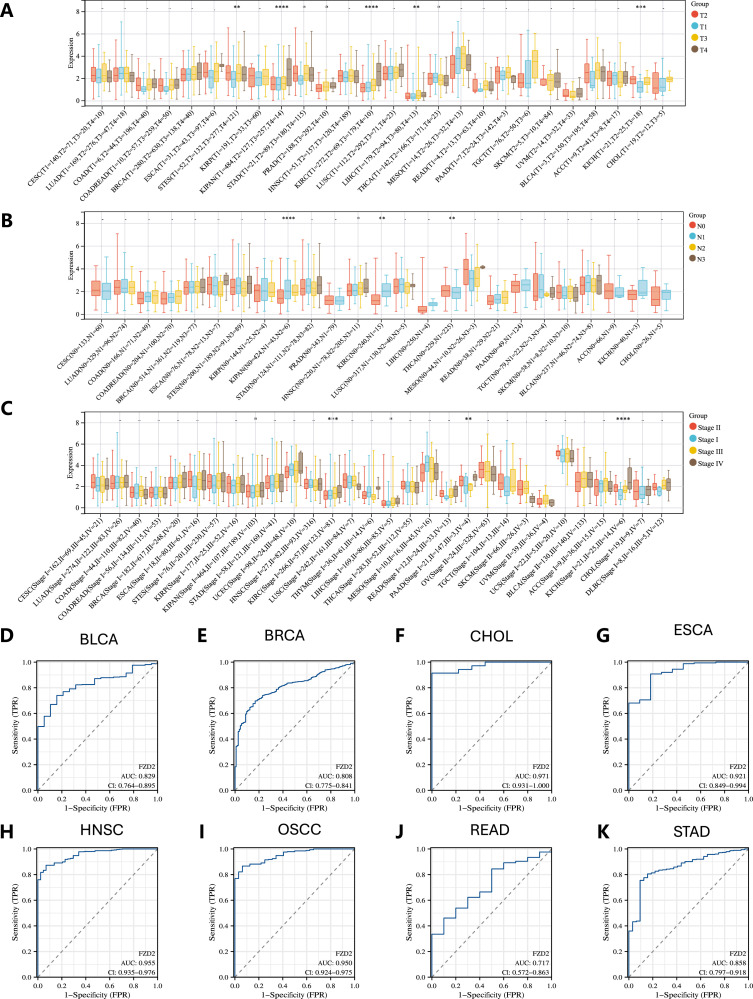
Clinical and diagnostic significance of FZD2 in pan-cancer. **(A)** Association between FZD2 expression and T stage across various cancers. **(B)** Relationship between FZD2 expression and N stage across different cancers. **(C)** Link between FZD2 expression and overall cancer stage in pan-cancer. **(D–K)** ROC curve analysis of FZD2’s diagnostic performance across eight cancer types using TCGA data. *P < 0.05, **P < 0.01, ***P < 0.001, ****P < 0.0001.

Analysis of TCGA data revealed a strong positive correlation between FZD2 expression and advanced cancer stages. Notably, significant differences in FZD2 expression were observed across T stages in eight cancers, including STES, KIPAN, STAD, PRAD, KIRC, LIHC, THCA, and KICH (Figure 3A). FZD2 expression was also associated with N stage in four cancers: KIPAN, HNSC, KIRC, and THCA (Figure 3B). Furthermore, FZD2 expression correlated with overall stage in KIPAN, KIRC, LIHC, PAAD, and KICH (Figure 3C). These findings underscore a significant link between FZD2 expression and clinical staging in multiple cancer types.

### 3.3 Diagnostic value of FZD2 in pan-cancer

The diagnostic potential of FZD2 across various cancer types was evaluated using ROC curve analysis, with the area under the curve (AUC) calculated to determine its predictive power. FZD2 showed promise as a diagnostic biomarker in BLCA, BRCA, CHOL, ESCA, HNSC, oral squamous cell carcinoma (OSCC), READ, and STAD, with AUCs of 0.829, 0.808, 0.971, 0.921, 0.955, 0.950, 0.717, and 0.858, respectively (Figures 3D–K). These ROC analyses support FZD2 as a robust diagnostic indicator in these cancers.

### 3.4 Overall survival (OS) and disease-free survival (DFS) analysis

**FIGURE 4:**
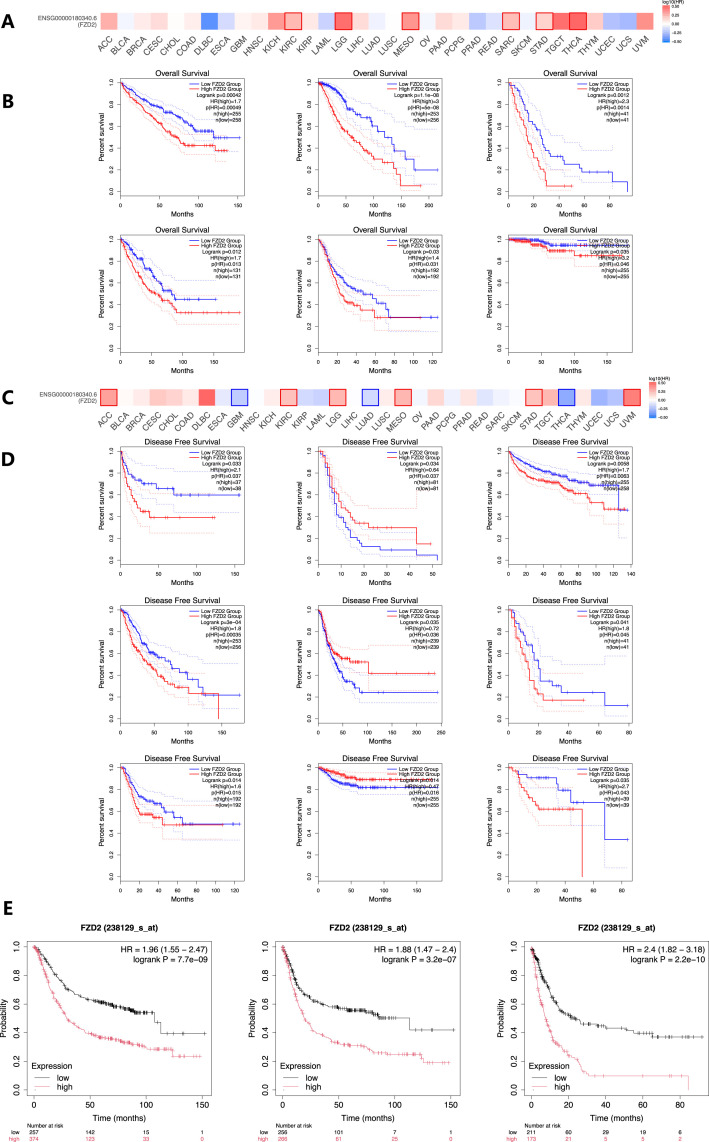
Prognostic significance of FZD2 mRNA expression in various cancers. **(A)** Overall survival (OS) analysis for FZD2 across 33 cancer types using the GEPIA database. **(B)** Kaplan–Meier survival curves comparing high and low FZD2 expression in six different cancer types. **(C)** Disease-free survival (DFS) analysis for FZD2 across 33 cancer types (GEPIA database). **(D)** Kaplan–Meier survival curves comparing high and low FZD2 expression in nine different tumors. **(E)** Prognostic value of FZD2 expression in STAD for OS, first progression (FP), and post-progression survival (PPS) (K-M plot).

Elevated FZD2 expression was associated with poor overall survival (OS) in cancers such as KIRC, LGG, MESO, SARC, and THCA (Figures 4A,B). Furthermore, analysis of disease-free survival (DFS) in the TCGA database indicated that higher FZD2 expression correlated with unfavorable outcomes in KIRC, LGG, MESO, STAD, and uveal melanoma (UVM) (Figures 4C,D), while lower FZD2 expression was linked to poorer DFS in GBM, LUAD, and THCA. The prognostic significance of FZD2 was further validated using Kaplan-Meier analysis in GEO datasets, where gastric cancer patients with elevated FZD2 mRNA levels exhibited worse prognoses compared to those with lower levels. Specifically, high FZD2 expression was identified as a risk factor for overall survival (OS), first progression (FP), and post-progression survival (PPS) in gastric cancer patients (P < 0.05 for each; Figure 4E). These findings underscore FZD2 mRNA expression as a prognostic marker in multiple cancers, particularly in gastric cancer.

### 3.5 FZD2 Co-Expression gene enrichment analysis and PPI network

**FIGURE 5:**
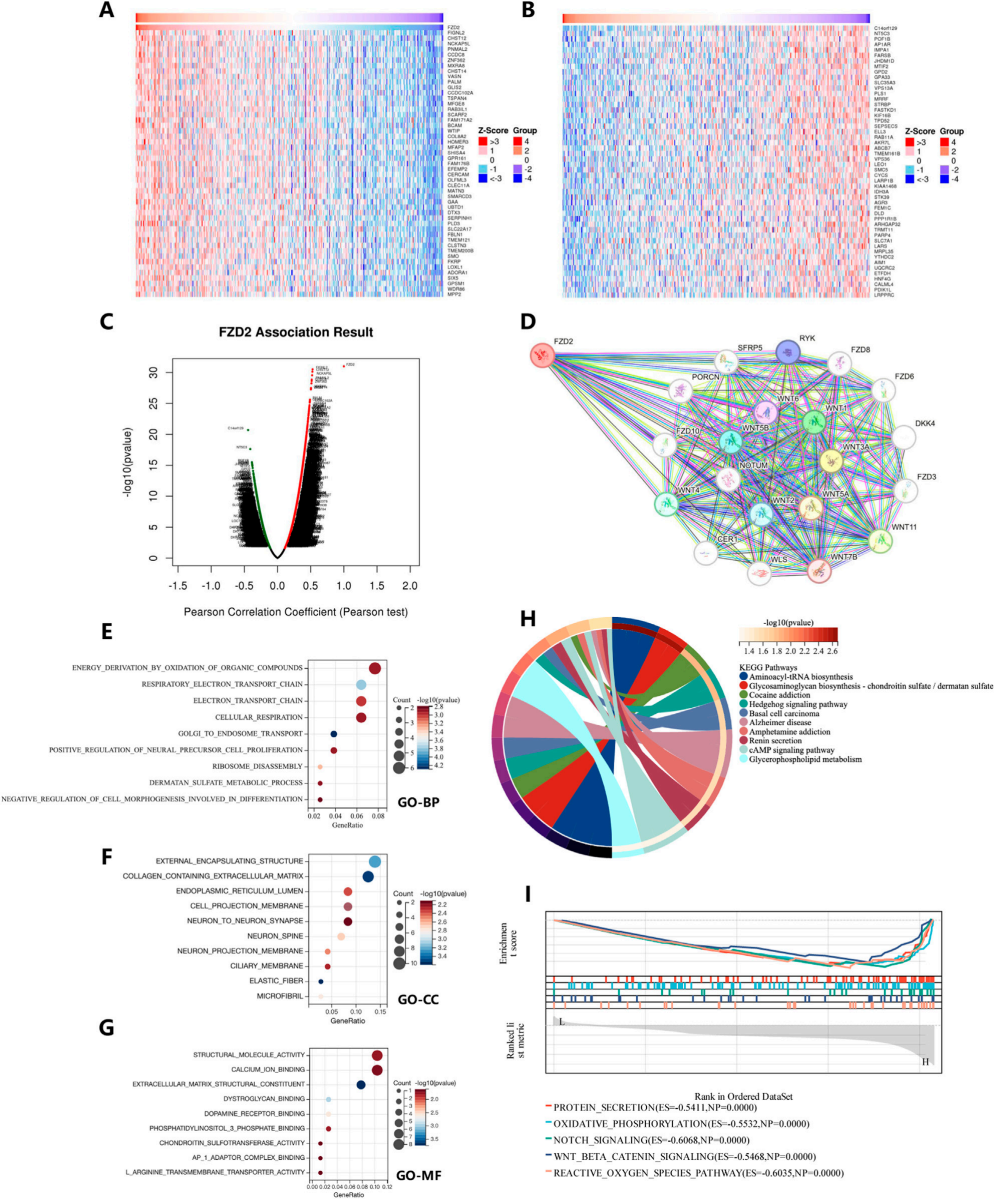
Gene function set enrichment analysis of FZD2 in STAD. **(A,B)** Heatmaps showing the top genes positively and negatively associated with FZD2 in STAD. **(C)** Scatter plot illustrating the associations of FZD2. **(D)** Protein-protein interaction (PPI) network analysis of FZD2. **(E–G)** Bubble charts presenting results from the GO pathway enrichment analysis in STAD. **(H)** Circle plot displaying the results of the KEGG pathway enrichment analysis in STAD. **(I)** GSEA enrichment analysis of FZD2.

To clarify FZD2’s potential functions in tumor tissues, we analyzed its co-expressed genes in STAD using the LinkedOmics database. Heat maps of the top 50 genes positively and negatively correlated with FZD2 are shown in Figures 5A,B, with additional association data in Figure 5C. To investigate potential protein interactions, we constructed an FZD2 protein-protein interaction (PPI) network using STRING (Figure 5D), identifying interactions with proteins involved in sphingolipid and ceramide metabolism, including WNT, SFRP, NOTUM, and other FZD family members. Gene Ontology (GO) and KEGG pathway analyses of genes closely related to FZD2 indicated primary associations with processes such as Golgi-to-endosome transport, collagen-containing extracellular matrix organization, basement membrane assembly, and extracellular matrix structural organization (Figures 5E–G). KEGG pathway enrichment analysis indicated that FZD2 is involved in aminoacyl-tRNA biosynthesis, glycosaminoglycan biosynthesis (chondroitin sulfate/dermatan sulfate), and cocaine addiction pathways (Figure 5H). In the HALLMARK gene set, associated pathways included protein secretion, oxidative phosphorylation, Notch signaling, Wnt/β-catenin signaling, and reactive oxygen species pathways (Figure 5I). These analyses suggest a positive correlation between FZD2 and Wnt signaling as well as biosynthesis pathways.

### 3.6 FZD2 mutation and RNA modification gene analysis

**FIGURE 6:**
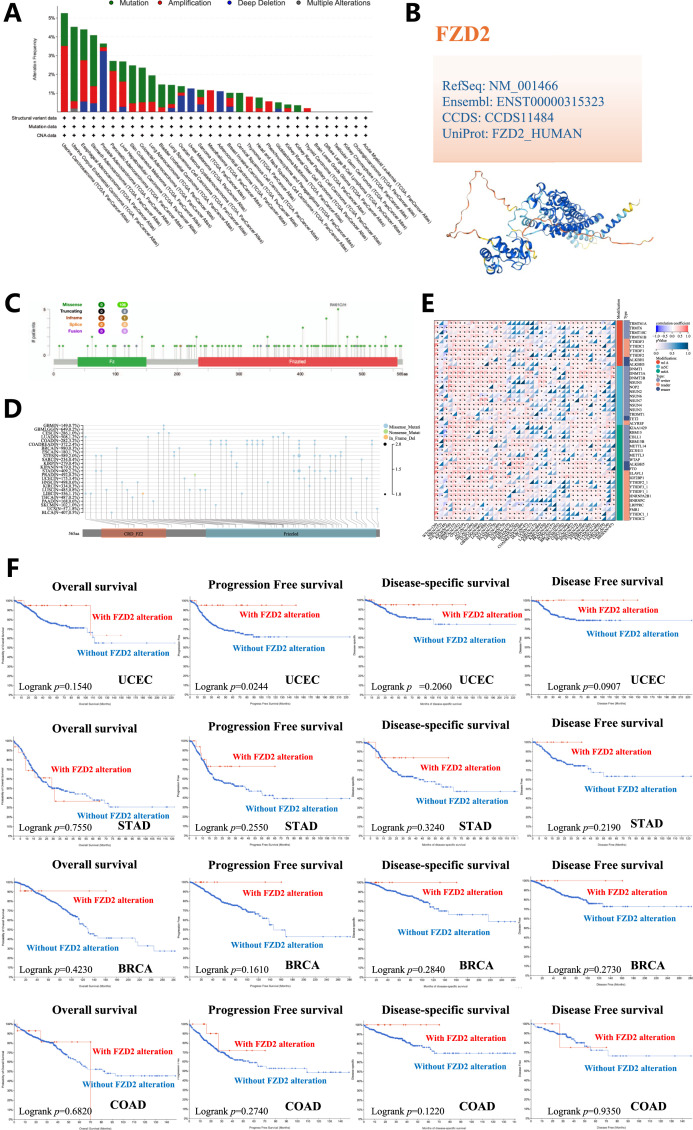
Genetic variation analysis. **(A)** Types of mutations observed in FZD2 across different tumor samples. **(B)** 3D representation of mutation sites with high mutation frequency. **(C)** Sites of mutations in FZD2. Multiple mutation sites were identified, with the R461C/H site in the bromodomain showing the highest alteration frequency. **(D)** Structural domain-specific mutation landscapes of FZD2 identified through pan-cancer analysis. **(E)** Pan-cancer analysis identifies significant correlations between 44 RNA modification genes and FZD2 expression. **(F)** Analysis of the correlation between FZD2 mutation status and overall, disease-specific, disease-free, and progression-free survival in UCEC, STAD, BRCA, and COAD.

Figure 6A, sourced from cBioPortal, illustrates the genetic alteration status of FZD2 across patient samples. The highest alteration frequency of FZD2 was observed in uterine carcinosarcoma (UCS), with the predominant alteration type being ‘amplification’ (copy number alteration). Notable alterations were also found in esophageal carcinoma (ESCA), pancreatic adenocarcinoma (PAAD), liver hepatocellular carcinoma (LIHC), mesothelioma (MESO), thymoma (THYM), and PCPG, particularly characterized by high copy number amplification. CNA of FZD2 was pronounced in uveal melanoma (UVM) and adrenocortical carcinoma (ACC), where copy number deletion accounted for all genetic alterations of FZD2 in those cancers. Figures 6B,C highlight numerous mutation sites in FZD2 within the TCGA cohort, focusing on the R461 C/H site in the bromodomain, which exhibited the highest frequency of alteration. Four TCGA samples (UCEC, STAD, BRCA, and COAD) were identified with mutations at this site. The D167G/Y alteration in FZD2 did not show a significant effect on clinical survival, except for progression-free survival in UCEC (Figure 6F). We also downloaded the Simple Nucleotide Variation dataset for level 4 of all TCGA samples processed by MuTect2 from GDC, integrating mutation data and obtaining structural domain information using the R package maftools (Figure 6D). Pan-cancer analysis revealed significant correlations between 44 RNA modification genes and FZD2 expression, with m6A regulators such as RBM15B and m1A regulators such as YTHDF1 positively associated with FZD2 in most tumors (Figure 6E).

### 3.7 Relationship between FZD2 expression and immune environment in human pan-cancer

**FIGURE 7:**
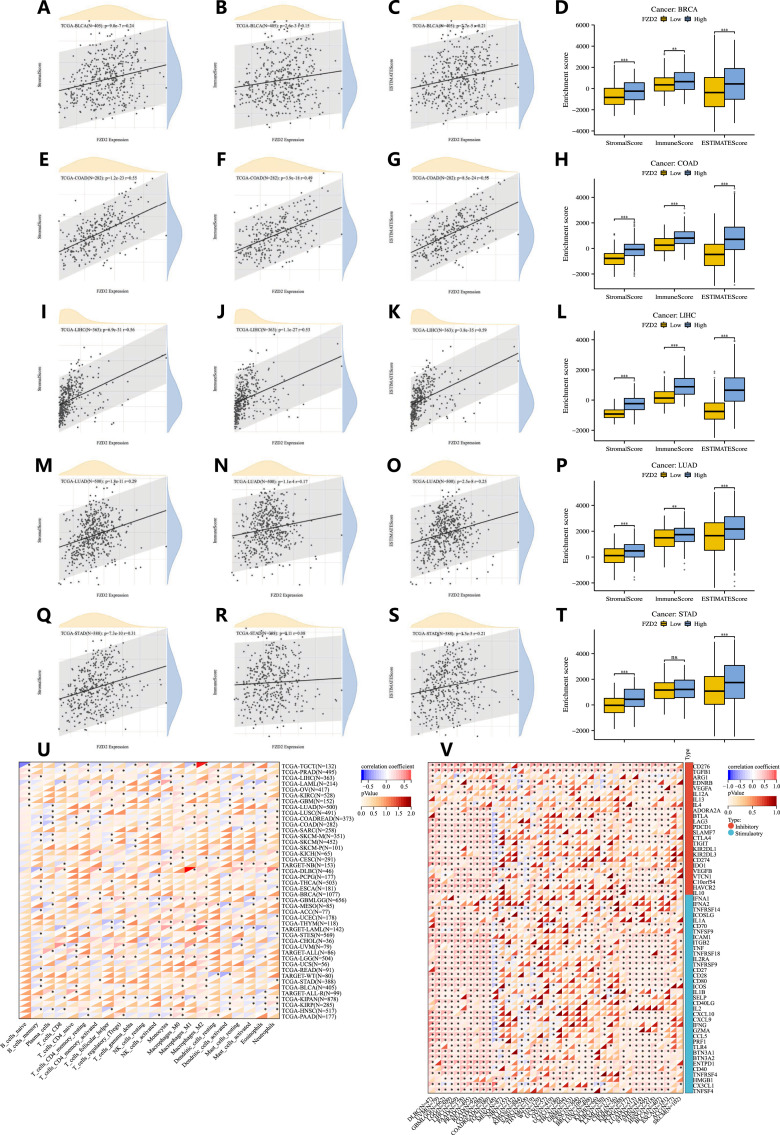
Correlation between FZD2 and immune cell infiltration in pan-cancer. **(A–T)** The relationship between FZD2 expression and immune scores, as well as differences in immune scores between high-FZD2 and low-FZD2 groups in BRCA, COAD, LIHC, LUAD, and STAD. **(U)** Correlation between FZD2 and the proportions of 22 infiltrating immune cell subsets in pan-cancer. **(V)** Correlation between FZD2 and 60 immune checkpoints in pan-cancer. *P < 0.05, **P < 0.01, ***P < 0.001, ****P < 0.0001.

**FIGURE 8:**
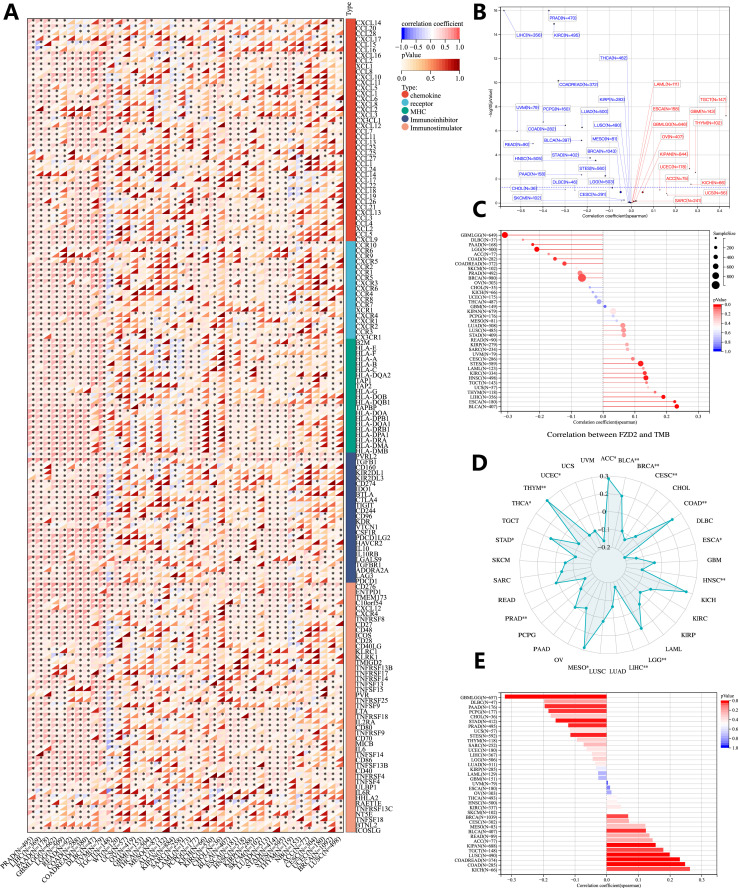
Genomic heterogeneity and gene expression analysis. **(A)** Correlation between FZD2 and immunomodulatory molecules. **(B)** Correlation of FZD2 expression with tumor purity. **(C)** Correlation of FZD2 expression with MATH. **(D)** Correlation of FZD2 expression with TMB. **(E)** Correlation of FZD2 expression with MSI. *P < 0.05, **P < 0.01, ***P < 0.001, ****P < 0.0001.

The ESTIMATE algorithm was applied to calculate stromal and immune scores as well as tumor purity. FZD2 expression showed a positive correlation with immune scores in BRCA, COAD, LIHC, LUAD, and STAD. Based on the median expression level, patients in these cancers were categorized into high and low FZD2 expression groups. Those in the high FZD2 group exhibited significantly higher immune, stromal, and ESTIMATE scores compared to the low-expression group (Figures 7A–T). Additionally, we analyzed the association between FZD2 expression and the abundance of 22 types of infiltrating immune cells across pan-cancer (Figure 7U). M2 macrophages showed the strongest correlation with FZD2 expression in the pan-cancer analysis. In STAD, FZD2 expression was correlated with memory B cells, activated memory CD4 T cells, M0 and M2 macrophages, and neutrophils. Figure 7V highlights the correlation between FZD2 expression and 60 genes associated with two immune checkpoint pathways, the majority of which were significantly correlated. Figure 8A demonstrates the association between FZD2 expression and immunomodulators, with FZD2 positively correlating with numerous immunomodulatory genes across pan-cancer. Notably, in STAD, FZD2 was positively associated with CTLA4, PDCD1, and LAG3. These findings suggest a potential role for FZD2 in modulating immune cell infiltration in diverse cancer types.

### 3.8 Genomic heterogeneity and FZD2 expression analysis

We investigated the association between FZD2 expression and genomic heterogeneity metrics—including tumor purity, mutant-allele tumor heterogeneity (MATH), tumor mutational burden (TMB), and MSI-as potential biomarkers for immunotherapy response and prognosis. FZD2 mRNA expression was correlated with these factors across 33 cancer types to assess how gene mutations might influence FZD2 expression. A scatter plot analysis revealed a significant positive correlation between FZD2 and tumor purity in three cancers (GBM, THYM, TGCT) and a negative correlation in 16 cancers, including LUAD, COAD, BRCA, STES, STAD, and PRAD (Figure 8B). Figure 8C demonstrates a strong positive association between FZD2 and MATH in ESCA, STES, HNSC, LIHC, and BLCA, whereas negative correlations were observed in cancers such as GBMLGG, LGG, COAD, and BRCA. A radar plot showed significant positive correlations between FZD2 and TMB in ACC, BLCA, COAD, LGG, MESO, and THYM, while negative correlations were observed in BRCA, CESC, ESCA, HNSC, LIHC, and PRAD (Figure 8D). Additionally, FZD2 expression was significantly associated with MSI in 16 tumor types (Figure 8E). These results suggest that FZD2 expression is closely linked to TMB and MSI, potentially impacting immunotherapy outcomes and prognostic evaluations.

### 3.9 Single-cell analysis of FZD2

**FIGURE 9:**
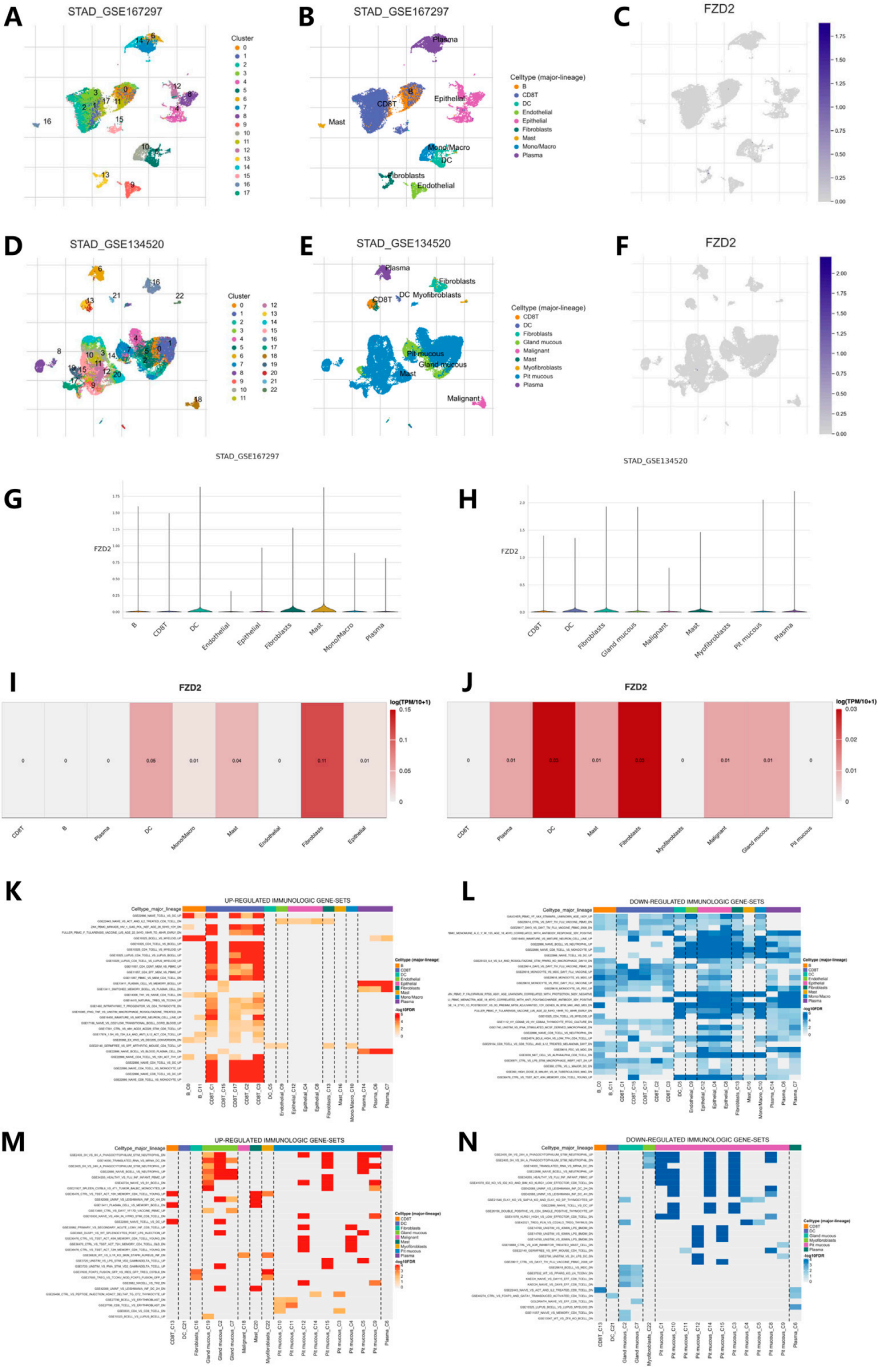
Single-cell analysis of FZD2 in STAD from the TISCH2 database. **(A–F)** UMAP plot visualizations of FZD2 distribution among different clusters and cell types. **(G–J)** Violin plots showing FZD2 expression across various cell types in STAD. **(K,L)** Upregulated and downregulated immunogenomes analyzed by GSEA enrichment of GSE167297. **(M,N)** Upregulated and downregulated immunogenomes analyzed by GSEA enrichment of GSE134520.

FZD2 expression in the STAD single-cell dataset was analyzed using TISCH2. Two single-cell datasets (GSE167297 and GSE134520)from STAD were annotated using Uniform Manifold Approximation and Projection (UMAP) for dimensionality reduction (Figures 9A,B,D,E). The datasets included various cell types such as CD8 T cells, dendritic cells, fibroblasts, mucous gland cells, malignant cells, mast cells, myofibroblasts, pit mucous cells, endothelial cells, epithelial cells, macrophages, and plasma cells. Figure 9C,F show the distinct FZD2 expression patterns across these immune cell types. Violin plots illustrating FZD2 expression revealed high levels in mast cells and fibroblasts (Figures 9G–J), while CD8^+^ T cells, B cells, plasma cells, and malignant cells showed notably lower expression. Analysis of two GEO datasets further confirmed that FZD2 is highly expressed in fibroblasts. Figures 9K–N present the results of GSEA enrichment analysis, showing up- and downregulated immunogenes in these gastric cancer datasets.

### 3.10 Drug sensitivity analysis and FZD2 expression

**FIGURE 10:**
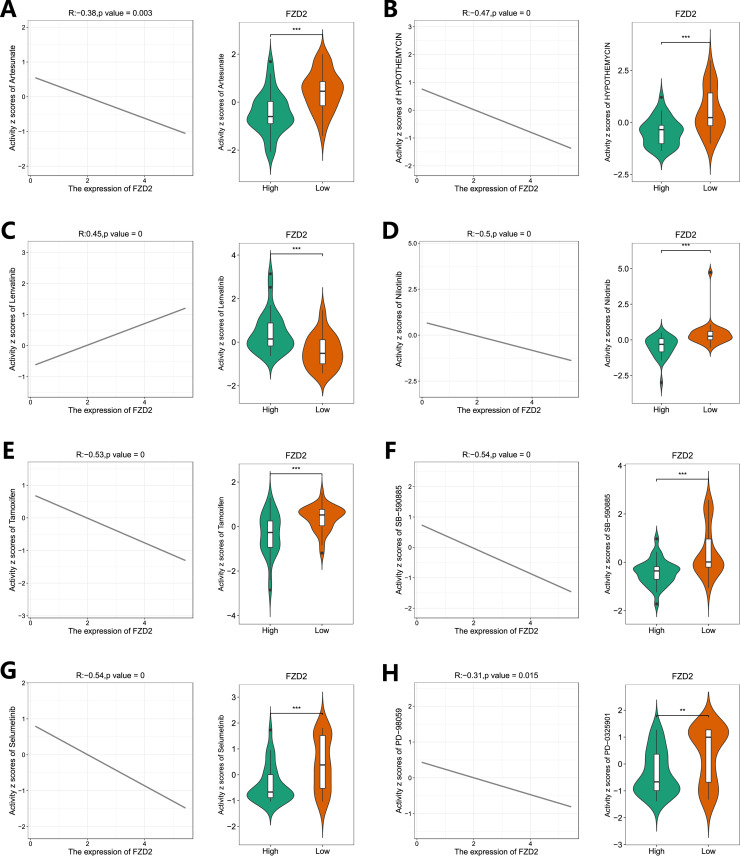
Drug sensitivity analysis of FZD2 in gastric cancer based on the TCGA database. The “pRRophetic” package was used to predict the sensitivity of STAD patients to eight different drugs **(A–H)**. *P < 0.05, **P < 0.01, ***P < 0.001, ****P < 0.0001.

Using the R package pRRophetic, drug sensitivity analysis was performed by correlating FZD2 gene expression with drug response data. We assessed responses to several drugs, including Artesunate, HYPOTHEMYCIN, Lenvatinib, Nilotinib, Tamoxifen, SB−590885, Selumetinib, and PD−98059, across varying FZD2 expression levels (Figures 10A–C). The low FZD2 expression group exhibited greater sensitivity to Artesunate, HYPOTHEMYCIN, Nilotinib, Tamoxifen, SB−590885, Selumetinib, and PD−98059, whereas the high FZD2 expression group showed enhanced sensitivity to Lenvatinib (Figures 10D–H). These findings suggest that FZD2 may be a promising therapeutic target in STAD.

### 3.11 Distribution of FZD2 in organelles and expression of FZD2 in clinical samples

**FIGURE 11:**
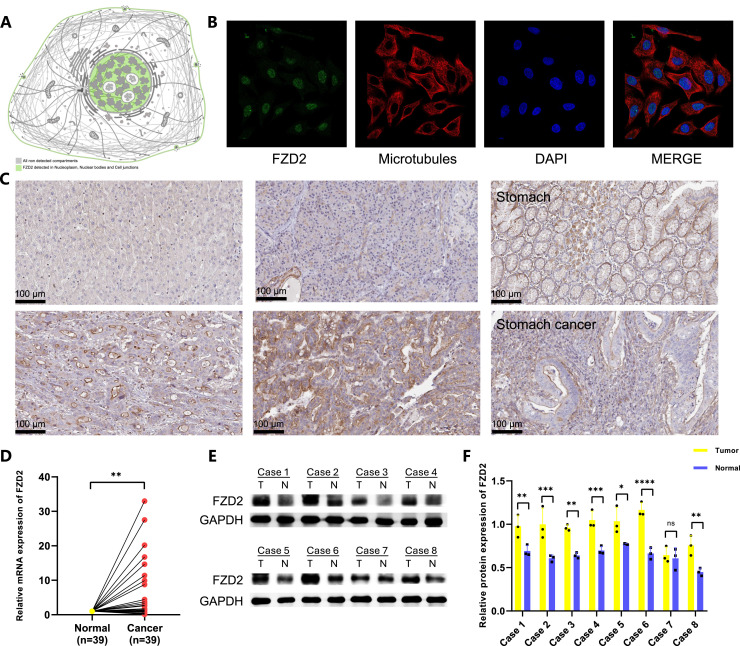
Expression of FZD2 in tumor is higher than that in normal tissue. **(A)** Detection of FZD2 in various cellular compartments. **(B,C)** Immunofluorescence and immunohistochemical images of FZD2 from the Human Protein Atlas HPA. **(D)** RT-qPCR validation of FZD2 expression in paired gastric cancer tissues. **(E,F)** Western blot analysis confirming FZD2 expression in paired gastric cancer tissues. (n = 3 biological replicates.) *P < 0.05, **P < 0.01, ***P < 0.001, ****P < 0.0001.

The HPA database revealed that FZD2 is present in various cellular compartments (Figure 11A), with increased FZD2 protein levels found in liver, pancreatic, and gastric cancer tissues compared to normal tissues (Figure 11C). Pan-cancer analysis of TCGA and GTEx cohorts showed elevated FZD2 mRNA expression across multiple cancer types. Immunofluorescence staining confirmed that FZD2 protein is primarily localized in the nucleoplasm and nuclear membrane (Figure 11B). RT-qPCR validation of FZD2 mRNA expression in 39 paired gastric cancer tissues (Figure 11D) demonstrated higher expression in tumor tissues compared to normal tissues. Western blot analysis of 8 paired gastric cancer samples further supported these results, showing increased FZD2 levels in tumor tissues (Figures 11E,F). Both RT-qPCR and Western blot analyses consistently showed elevated FZD2 expression in tumor tissues.

### 3.12 Knockdown of FZD2 inhibited gastric cancer cell migration, proliferation, and invasion in vitro

**FIGURE 12:**
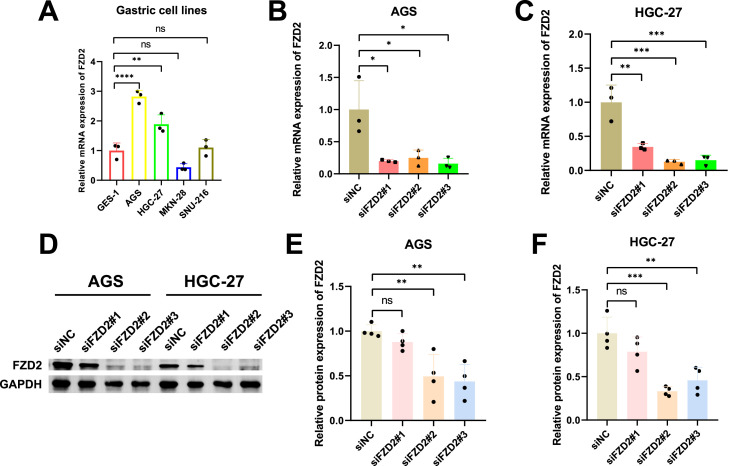
Cell line selection and siRNA validation. **(A)** FZD2 mRNA expression levels in various gastric cell lines (n = 3 biological replicates). **(B,C)** RT-qPCR analysis confirming the siRNA-mediated knockdown of FZD2 in AGS and HGC-27 cell lines (n = 3 biological replicates). **(D–F)** Western blot results validating the efficiency of FZD2 knockdown (n = 4 biological replicates). *P < 0.05, **P < 0.01, ***P < 0.001, ****P < 0.0001.

**FIGURE 13:**
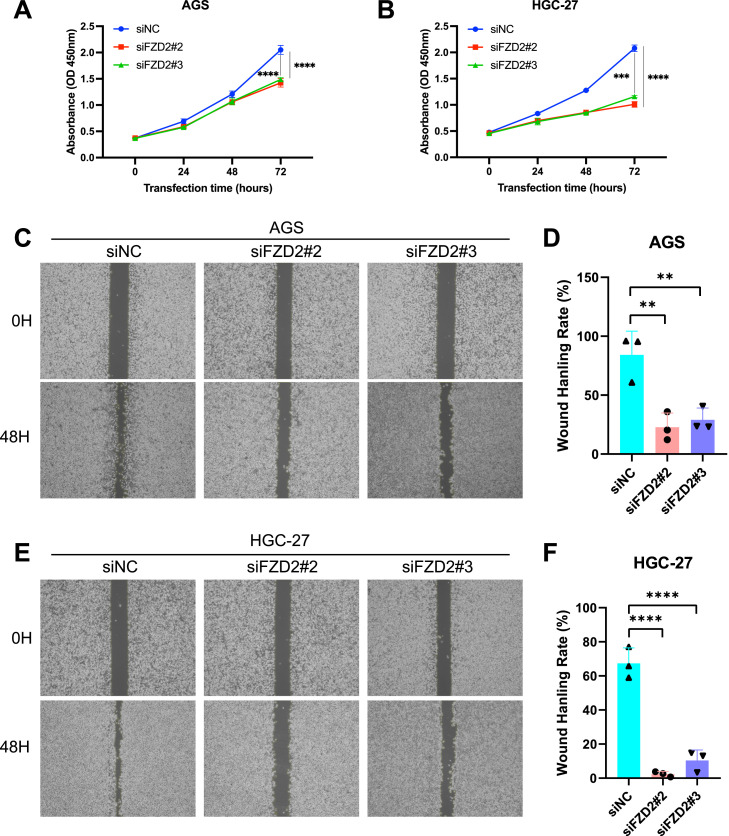
FZD2 knockdown inhibits the proliferation, migration, and invasion while promotes the apoptosis of gastric cancer cell. **(A,B)** CCK-8 assay evaluate the effect of FZD2 knockdown on gastric cancer cell proliferation. **(C–F)** Wound healing assay assessing the migration ability of FZD2 knockdown cell lines. Data are presented as mean ± SD from three independent experiments. (n = 3 biological replicates.) *P < 0.05, **P < 0.01, ***P < 0.001, ****P < 0.0001.

**FIGURE 14:**
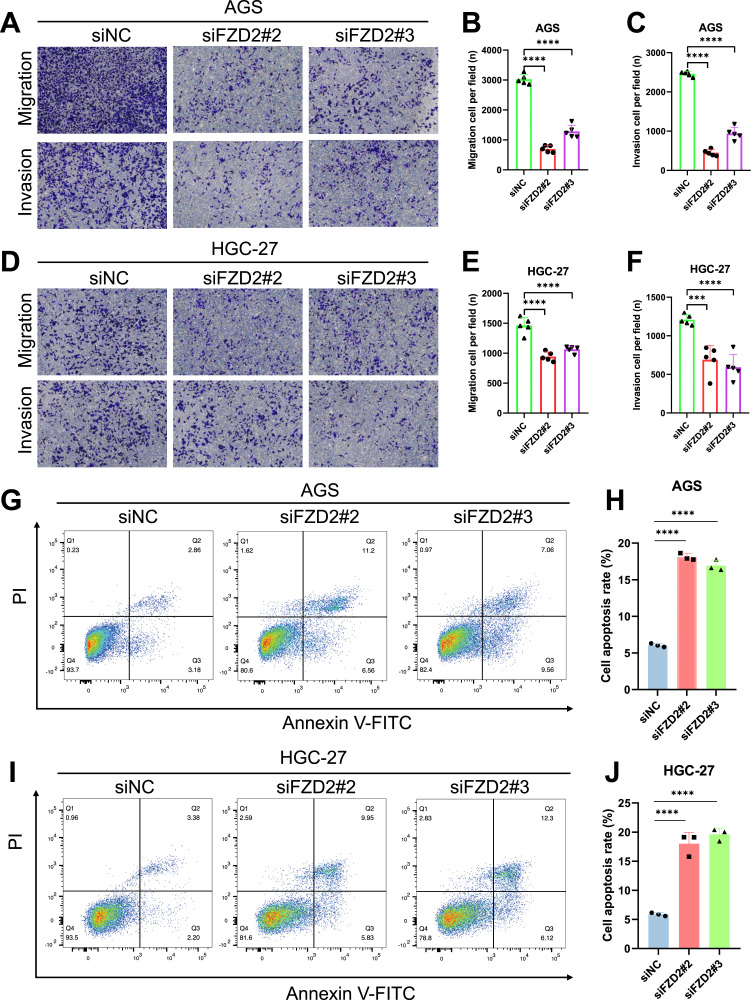
Evaluation of the potential role of FZD2 using transwell assays and apoptosis analysis. **(A–F)** Transwell assays assessing the migration and invasion capabilities of knockdown cell lines (n = 5 biological replicates). **(G–J)** Flow cytometry analysis of apoptosis following siRNA-mediated knockdown of FZD2 in AGS and HGC-27 cell lines. Data are presented as mean ± SD of three experiments (n = 3 biological replicates). *P < 0.05, **P < 0.01, ***P < 0.001, ****P < 0.0001.

FZD2 expression was found to be elevated in AGS and HGC-27 cells, but lower in MKN-28 cells (Figure 12A). To explore the role of FZD2 in gastric cancer progression, loss-of-function experiments were performed in STAD cell lines. RT-qPCR and Western blot analysis confirmed the efficient knockdown of FZD2 in AGS and HGC-27 cells (Figures 12B–F). CCK-8 assays revealed that FZD2 silencing inhibited cell proliferation (Figures 13A,B). Wound healing assays showed that FZD2 knockdown impaired the migration capacity of STAD cell lines (Figures 13C–F). Moreover, Transwell migration and invasion assays demonstrated a significant reduction in both migration and invasion in FZD2-depleted gastric cells (Figures 14A–F). Additionally, apoptosis assays showed that silencing FZD2 increased the apoptotic rate in gastric cancer cells (Figures 14G–J).

### 3.13 Knockdown of FZD2 suppresses gastric cancer cell growth in vivo

**FIGURE 15:**
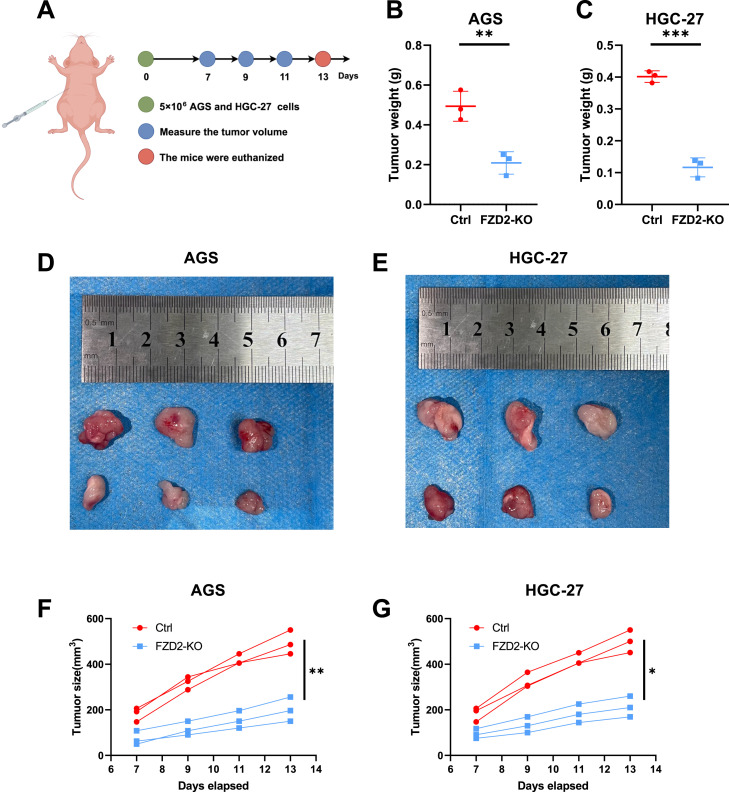
FZD2 knockdown inhibits AGS and HGC-27 gastric cancer growth in vivo. **(A)** CDX models were established (n = 3 per group). **(B,C)** Quantification of tumor weights in the control and FZD2 KO groups at the endpoint. **(D,E)** Representative images of tumors extracted from the control and FZD2 KO groups at the experimental endpoint. **(F,G)** Growth curves of xenograft tumors in nude mice injected with control or FZD2-knockout (KO) cells. Tumor volumes were measured at the indicated time points post-injection. (n = 3 biological replicates.) *P < 0.05, **P < 0.01, ***P < 0.001, ****P < 0.0001.

To further validate our *in vitro* findings, we performed *in vivo* experiments using human AGS and HGC-27 xenografts. A total of 5 × 10^6^ FZD2 knockout (KO) AGS and HGC-27 gastric cancer cells were resuspended in sterile PBS and mixed with Ceturegel^®^ matrix gel before being subcutaneously injected subcutaneously into the flank area of 6–7-week-old male BALB/c-nude mice (n = 3 per group). Tumor growth was monitored, and when the tumor volume reached or exceeded 1 × 10^3^ mm^3^, the mice were euthanized for further analysis. The results demonstrated that FZD2 KO significantly suppressed tumor growth and reduced tumor size (Figures 15A–D).

## 4 Discussion

Our study represents the first systematic pan-cancer analysis of FZD2, revealing its dual roles as a prognostic biomarker and molecular driver across 33 cancer types. Unlike single-cancer studies, we uncovered FZD2’s context-dependent functions in both canonical Wnt activation (e.g., Wnt3A/ROR2 axis) and immune evasion mechanisms (e.g., M2 macrophage recruitment). This unified framework reconciles prior conflicting findings and highlights FZD2’s potential to modulate tumorigenesis through multiple pathways. FZD2’s widespread overexpression in BLCA, BRCA, CHOL, and STAD aligns with prior studies linking Wnt/β-catenin signaling activation to oncogenic processes, including proliferation, epithelial-mesenchymal transition (EMT), and metastasis (Fu et al., 2020; Ning et al., 2022; Meagher et al., 2024). Notably, Gujral et al. demonstrated that Wnt signaling *via* FZD receptors enhances cell growth and migration in colorectal cancer, underscoring the potential oncogenic role of FZD2 in diverse cancer types (Gujral et al., 2014). These findings establish FZD2 as a pan-cancer biomarker and molecular driver, but their functional implications require systematic dissection. The Wnt/β-catenin signaling pathway plays a critical role in cellular processes like proliferation, differentiation, migration, and tissue polarity (Zhan et al., 2017). Dysregulation of this pathway, particularly its abnormal activation, results in β-catenin accumulation, leading to uncontrolled cell growth and tumor development. As key receptors in this pathway, FZDs, particularly FZD2, activate downstream signaling by binding to Wnt ligands, leading to tumorigenesis (Milovanovic et al., 2004; Fu et al., 2020). Specifically, FZD2 has been recognized as a critical receptor in the non-canonical Wnt pathway and is highly expressed across various cancers, where it serves as a marker of poor prognosis (Han et al., 2022). For instance, FZD2 has been shown to bind Wnt3A, which is activated by ROR2 molecules, initiating the canonical Wnt pathway and promoting oncogenesis in lung tissue (Li et al., 2008).

Our differential expression analysis revealed significant upregulation of FZD2 in cancers such as bladder cancer (BLCA), breast cancer (BRCA), cholangiocarcinoma (CHOL), and STAD, while it was downregulated in kidney cancers (KICH, KIRC). This tissue-specific expression pattern aligns with the findings of Wang et al., who reported elevated FZD2 levels in HCC, correlating with advanced tumour stages and poor prognosis (Liu et al., 2021). In gastric cancer (GC), gene expression profiling highlighted FZD2’s significant role in tumorigenesis. Furthermore, recent studies indicate that FZD2 is more highly expressed in HCC tissues compared to adjacent normal tissues, with higher FZD2 levels associated with lower recurrence-free survival rates (Liu et al., 2021). Additionally, FZD2 expression is linked to the mesenchymal phenotype in liver cancer cells, driving increased migration and invasiveness (Asano et al., 2017). Similarly, FZD2 has been shown to promote migration and invasion in oral squamous cell carcinoma (OSCC) through the STAT3 pathway (Zhang et al., 2015). Beyond expression patterns, FZD2’s oncogenic activity is mediated through both canonical Wnt signaling (e.g., Wnt3A/ROR2 axis) and non-canonical pathways (e.g., TGF-β/EMT induction), suggesting context-dependent regulatory networks. Single-cell analysis further underscored the overexpression of FZD2 in fibroblasts within the tumor microenvironment. This is significant, as fibroblasts play a crucial role in tumor immunity and matrix remodeling, facilitating metastasis (Kasashima et al., 2023). In ovarian cancer, FZD2 has been implicated in extracellular matrix remodeling, a process critical for enabling metastasis (Luo et al., 2019). Our protein-protein interaction (PPI) and co-expression analyses corroborate these findings, demonstrating FZD2’s interaction with proteins involved in Wnt signaling and sphingolipid metabolism, which underscores its multifaceted role in cancer biology. Further, gene set enrichment analysis revealed that FZD2 is associated with critical pathways such as Notch signaling and oxidative phosphorylation, both of which are essential for cancer cell survival and proliferation.

Kaplan-Meier survival analysis confirmed the prognostic value of FZD2, showing that high FZD2 expression correlates with unfavorable overall survival (OS) and disease-free survival (DFS) in multiple cancers, particularly in STAD. This supports the findings of Astudillo et al., who observed that FZD2 overexpression correlates with poor prognosis in gastrointestinal cancers (Astudillo, 2021). Given its correlation with survival outcomes, FZD2 may serve as a valuable biomarker for stratifying patients for targeted therapies. Our analysis also revealed that elevated FZD2 expression is positively correlated with immune cell infiltration, immune scores, and immune checkpoint gene expression, including CTLA4 and PDCD1 (PD-1). This is consistent with findings by Huang et al., which suggest that FZD2 modulates the immune microenvironment by affecting immune checkpoint pathways (Huang et al., 2022). Notably, FZD2 expression was strongly correlated with M2 macrophage infiltration, suggesting that FZD2 may contribute to immune evasion, creating an immunosuppressive microenvironment conducive to tumor growth (Wang et al., 2015). These findings have important implications for cancer immunotherapy, as FZD2 expression may impact the efficacy of immune checkpoint inhibitors. The translation of FZD2’s dual roles into clinical practice requires addressing three core challenges: determining the molecular basis for its ligand specificity, validating bromodomain inhibitors as synergistic agents with PD-1 blockade in hypermutated tumors, and establishing the FZD2-TMB/MSI signature as a predictive biomarker for Wnt-targeted therapies in advanced gastric cancer. Addressing these questions will bridge the gap between basic research and clinical implementation.

In addition to immune cell infiltration, our study found that FZD2 expression correlates with genomic heterogeneity indicators, such as TMB and MSI, both of which are markers associated with improved responses to immune checkpoint inhibitors (ICIs). Our findings demonstrate a positive correlation between FZD2 expression and TMB in cancers such as colorectal adenocarcinoma (COAD), supporting the hypothesis that FZD2 targeting could improve ICI efficacy, especially in tumors with high TMB. Previous research has shown that frameshift mutations in Wnt pathway regulators like AXIN2 and TCF7L2 are common in gastric cancers with high MSI, further linking Wnt pathway dysregulation to tumor progression (Kim et al., 2009).

FZD2’s involvement in cancer stem cells (CSCs) also complicates its role in tumor biology. CSCs are a subpopulation of tumor cells with self-renewal capability and high resistance to chemotherapy and radiotherapy, contributing to recurrence and metastasis (Li et al., 2008). The Wnt/β-catenin pathway regulates the self-renewal of liver CSCs, and several FZD family members are associated with CSC functions and drug resistance (Tammela et al., 2017). For example, FZD7 regulates stem cell activity in the gastric and intestinal epithelium, with its expression increasing in gastric cancer cells (Flanagan et al., 2017). Similarly, our study indicates that FZD2 may regulate tumor cell stemness through Wnt signaling, potentially contributing to chemoresistance. FZD2 expression was also correlated with drug sensitivity profiles; as FZD2 expression upregulated, cell sensitivity to drugs such as cobimetinib, selumetinib, bafetinib, and tamoxifen decreased, indicating that FZD2 may influence chemoresistance. Our data suggest that FZD2 could serve as a predictive biomarker for chemotherapy resistance in gastric cancer. Clinically, this could enable stratified treatment strategies: patients with high FZD2 levels might benefit from upfront combination therapy with FZD2 inhibitors and taxanes, while low-FZD2 patients could receive standard-of-care regimens. Such precision approaches would reduce unnecessary toxicities and improve survival outcomes.

In functional assays using gastric cancer cell lines, FZD2 knockdown significantly inhibited cellular proliferation, migration, and invasion. Specifically, CCK8 assays revealed dramatic reductions in cell viability, while wound healing and Transwell assays demonstrated markedly diminished migratory and invasive capacities. These findings collectively suggest that FZD2 promotes oncogenic phenotypes in gastric cancer, potentially through TGF-β-induced EMT, as supported by prior studies linking FZD2 to non-canonical Wnt signaling pathways. To validate this *in vivo*, we established subcutaneous xenograft models using FZD2-silenced AGS cells. The results consistently mirrored our *in vitro* observations: tumors formed by FZD2-deficient cells exhibited smaller volumes compared to controls. While current validation is limited to gastric cancer models, future studies aim to expand the scope using organoid technology to address tumor heterogeneity. Furthermore, CRISPR-based screens and PDX model studies combined with immunotherapy will elucidate FZD2’s role in immune regulation and therapeutic resistance. This work provides a critical foundation for developing FZD2-targeted combinatorial therapies in oncology.

Our study underscores the critical role of FZD2 in cancer biology, highlighting its potential as both a prognostic biomarker and a therapeutic target, while also laying the groundwork for precision oncology approaches that target its multifaceted functions. The impact of FZD2 on immune cell infiltration, tumor mutational burden (TMB), microsatellite instability (MSI), and drug resistance mechanisms supports its relevance in precision oncology. While this study establishes the multifaceted functions of FZD2, several key questions remain. The interaction between FZD2 and non-canonical Wnt ligands, such as Wnt5a, may vary across different cancer types, influencing its functional role in tumor progression. From a therapeutic perspective, FZD2 inhibitors, including small molecules targeting its bromodomain, may have the potential to enhance anti-tumor immunity when combined with immune checkpoint inhibitors (ICIs). Additionally, FZD2-based signatures, particularly in combination with TMB and MSI, may provide superior prognostic value compared to existing models. Addressing these issues will be critical for advancing precision oncology and improving patient outcomes through personalized FZD2-targeted strategies. Further investigations into FZD2’s interaction with immune checkpoints and its role in the tumor microenvironment will be invaluable for developing targeted therapies, especially in immune-resistant cancers.

## 5 Conclusion

In summary, our study identifies FZD2 as a significant pan-cancer biomarker, particularly in gastric cancer, where it may serve as a target for therapeutic interventions. The robust associations between FZD2 expression and clinical outcomes, coupled with its functional implications in promoting tumor progression and influencing immune responses, underscore the potential of FZD2 in personalized medicine. Future investigations should focus on elucidating the specific molecular mechanisms by which FZD2 contributes to cancer progression and exploring its application in targeted therapies. The findings from our functional assays further support the importance of FZD2 in cancer biology, paving the way for more effective treatment strategies and improved patient outcomes in the future.

## Data Availability

The original contributions presented in the study are publicly available. This data can be found here: TCGA (https://www.cancer.gov/tcga), cBioPortal (https://www.cbioportal.org), GEO (https://www.ncbi.nlm.nih.gov/geo, e.g., GSE134520, GSE167297), and The Human Protein Atlas (https://www.proteinatlas.org).
